# A blinded randomised placebo-controlled trial investigating the efficacy of morphine analgesia for procedural pain in infants: Trial protocol

**DOI:** 10.12688/wellcomeopenres.10005.2

**Published:** 2017-01-26

**Authors:** Rebeccah Slater, Caroline Hartley, Fiona Moultrie, Eleri Adams, Ed Juszczak, Richard Rogers, Jane E Norman, Chetan Patel, Kayleigh Stanbury, Amy Hoskin, Gabrielle Green, Poppi Trial Team

**Affiliations:** 1 Department of Paediatrics, University of Oxford, Oxford, UK; 2 National Perinatal Epidemiology Unit (NPEU), University of Oxford, Oxford, UK; 3 Nuffield Department of Anaesthetics, John Radcliffe Hospital, Oxford, UK; 4 MRC Centre for Reproductive Health, The Queen’s Medical Research Institute, Edinburgh, UK; 5 Oxford Eye Hospital, John Radcliffe Hospital, Oxford, UK

**Keywords:** Infant, Pain, Morphine, Brain, EEG, Retinopathy of Prematurity, Analgesia

## Abstract

Infant pain has both immediate and long-term negative consequences, yet in clinical practice it is often undertreated. To date, few pain-relieving drugs have been tested in infants. Morphine is a potent analgesic that provides effective pain relief in adults, but there is inconclusive evidence for its effectiveness in infants. The purpose of this study is to establish whether oral morphine provides effective analgesia for procedural pain in infants.

A blinded, placebo-controlled, parallel-group randomized, phase II, clinical trial will be undertaken to determine whether morphine sulphate administered orally prior to clinically-required retinopathy of prematurity (ROP) screening and heel lancing provides effective analgesia.  156 infants between 34 and 42 weeks’ gestational age who require a clinical heel lance and ROP screening on the same test occasion will be included in the trial. Infants will be randomised to receive either a single dose of morphine sulphate (100 μg/kg) or placebo. Each infant will be monitored for 48 hours and safety data will be collected during the 24 hours following drug administration.

The primary outcome will be the Premature Infant Pain Profile–revised (PIPP-R) score during the 30 second periods after ROP screening. The co-primary outcome will be the magnitude of nociceptive-specific brain activity evoked by a clinically-required heel lance. Infant clinical stability will be assessed by comparing the number of episodes of bradycardia, tachycardia, desaturation and apnoea, and changes in respiratory support requirements in the 24-hour periods before and after the clinical intervention. In addition, drug safety will be assessed by considering the occurrence of apnoeic and hypotensive episodes requiring intervention in the 24-hour period following drug administration. This study has been published as an *Accepted Protocol Summary *by *The Lancet*.

## Background and rationale

### Context

The Protocol for our clinical trial titled ‘A blinded randomised placebo-controlled trial investigating the efficacy of morphine analgesia for procedural pain in infants’ is presented here.

A brief summary of the Trial Protocol has already been published by *The Lancet* as an *Accepted Protocol Summary* ^1^. The full Protocol was reviewed by The Lancet Editorial Board and three external reviewers, with a view to publishing the results once data collection is complete ^2, 3^. Following *The Lancet* review process key changes were made to the Protocol (Amendment 1) and prior to the recruitment further amendments were made following review by the Medicines and Healthcare products Regulatory Authority (MHRA) (Amendment 2) and following a change in the drug manufacturer (Amendment 3 & 4).

### Background

Pain in infancy is a serious clinical issue. While infant pain is recognised to have both immediate and long-term consequences, it is still drastically under-treated in this population. Although an infant receiving intensive care will experience an average of 10 painful procedures per day, specific analgesia is provided less than 25% of the time ^4^. Furthermore, this provision of analgesia includes ‘comfort techniques’ (such as skin-to-skin maternal contact) and does not specifically refer to pharmacological analgesia, which would be routinely used in older children and adults ^4^.

Despite the high burden of pain in the neonatal population, less than 40% of UK neonatal units have a Pain Management Protocol for minor painful procedures ^5^. The historical misconception that infants do not ‘feel’ pain has led to the predisposition to under-treat pain in clinical practice ^6^. For example, surveys of neonatal practice demonstrate that 50% of neonatal units in the UK and US do not routinely provide premedication for highly invasive elective intubation, despite evidence of clinical benefit ^7, 8^. If infants are to receive the best possible care a change in clinical practice is needed whereby there is an expectation to recognise and effectively treat infant pain. As such, it is essential that clinical trials that test the efficacy of analgesic medication be conducted using specific and sensitive measures of infant pain.

### Retinopathy of prematurity (ROP) screening

The most vulnerable infants, with the highest burden of pain, are those born prematurely who require intensive neonatal care. In the UK, newborn infants admitted to neonatal intensive care units (NICU) will be hospitalised for an average of 56 days, and the youngest and sickest infants will experience up to 50 painful procedures per day ^4, 9^. Retinopathy of Prematurity (ROP) screening is an example of a highly invasive medical procedure that is repeatedly performed on premature infants. ROP is a condition of the retinal vascular system that affects premature infants, which if untreated, can lead to permanent blindness. As such there is a national ROP screening programme, which involves regular ophthalmic screening of premature infants to identify whether ROP treatment is necessary. Unfortunately, ROP screening is considered to be painful ^10^, and is stressful for both infants and parents ^11^. Infants are extremely unsettled both during and after the procedure, and increased pain scores ^12^, increased salivary cortisol ^13^, and an increased likelihood of apnoeic episodes in the 48 hour period after screening have been reported ^12, 14^.

Pain management strategies that have been used for ROP screening include the use of topical local anaesthetic, sucrose and breast milk, but they have all been shown to be inadequate for reducing pain ^15– 17^. Indeed, a recent Cochrane review examining the use of local anaesthetic concluded that “screening remains a painful procedure and the role of non-pharmacological and pharmacological intervention including different local anaesthetic agents should be ascertained in future randomised trials” ^15^. The level of clinical distress caused by this procedure is exemplified in a recent study, which reports that in the 24-hour period following ROP screening 41% of infants have apnoeic episodes compared to only 19% before the examination ^12^.

It is plausible that the immediate distress caused to an infant during ROP screening and the resultant physiological instability that follows the examination may be alleviated if effective pain relief is provided during the procedure.

### Morphine analgesia

Morphine is a potent analgesic that provides effective pain relief for procedural pain in adults ^18^ but evidence for its effectiveness in infants is less clear. Morphine is commonly used on neonatal units as a sedative. For example, during mechanical ventilation intravenous morphine has been shown to reduce physiological instability; reduce behavioural responses; and improve ventilator synchrony ^19, 20^. Although morphine clearly provides effective sedation in infants, the efficacy of morphine as an analgesic remains controversial ^20– 22^. A recent Cochrane review concluded that there is insufficient evidence to recommend routine clinical use of morphine for procedural pain relief in mechanically ventilated infants ^23^. To date, no randomised controlled trials have been completed whereby morphine has been administered to healthy non-ventilated infants prior to acute painful procedures. Given the need for analgesics in the neonatal population and the limited availability of other analgesic drugs, it is essential that the efficacy of morphine analgesia be properly tested. It is plausible that administration of morphine prior to ROP screening may provide effective pain relief and consequently reduce the resultant physiological instability caused by the procedure.

To date only one trial has attempted to test whether morphine analgesia can reduce pain during ROP screening ^24^. While the results of this study suggested that morphine may provide effective pain relief, unfortunately, the trial was stopped prematurely due to changes to research regulations of the Medicines and Healthcare products Regulatory Authority (MHRA) and the need to obtain new approval to continue the study. The authors determined a sample size of 63 infants was needed, but were only able to recruit 18 infants in the study period and so did not reach the sample size necessary to detect whether morphine caused a significant reduction in pain scores ^24^.

### Clinical measures of pain in infants

Measures of pain in infants primarily rely on measuring changes in infant behaviour in response to noxious events. In premature infants the most validated clinical pain tool is the Premature Infant Pain Profile (PIPP) ^25, 26^. The PIPP primarily relies on the quantification of behavioural and physiological responses that are evoked by noxious stimulation. As PIPP is a composite multimodal measure, incorporating measures of heart rate, oxygen saturation and facial expression change, it allows for different aspects of the infant pain experience to be captured. It is well-validated for pain assessment in premature infants and has been widely used as the primary outcome measure for infant pain in many clinical trials ^27– 29^. If administration of morphine prior to ROP screening significantly reduced subsequent pain scores, there would be a strong rationale for its use in clinical practice.

### Measures of nociceptive-specific brain activity and reflex withdrawal activity

While behavioural measures are presently the gold standard for the assessment of pain, new electrophysiological techniques have more recently been developed to identify patterns of nociceptive brain and spinal cord activity in infants ^30, 31^. These objective and quantifiable neurophysiological measures have been used as the primary outcome measure in a recent clinical trial published in *The Lancet,* providing evidence to suggest that sucrose may not provide analgesia during clinical heel lancing ^32^. It would be highly advantageous to use these electrophysiological measures of nociceptive brain and spinal cord activity in clinical trials investigating the efficacy of morphine analgesia. This will provide a unique opportunity to gain a mechanistic insight into how morphine alters nociceptive activity in the immature infant central nervous system.

Measures of nociceptive brain activity evoked by ROP screening cannot be easily recorded due to difficulties in identifying the precise time when the nociceptive stimulus is applied. However, nociceptive-specific patterns of brain activity have been extremely well-characterised following clinical heel lancing ^30, 31, 33^. Heel lancing is a clinical procedure frequently performed in neonates to provide blood samples for the monitoring of jaundice, blood sugar, electrolytes and other haematological parameters. Due to the frequency of this procedure (which can be performed multiple times a day in some premature infants) it is feasible to arrange that ROP screening occurs immediately after a clinical blood sample is taken. The opportunity to perform ROP screening and a clinical heel lance on the same occasion means it is possible to administer morphine or a placebo in advance of both procedures. Therefore it is feasible to not only test whether morphine effectively reduces clinical pain scores during ROP screening but also to establish whether morphine reduces nociceptive brain and spinal cord activity evoked by a noxious heel lance.

### Benefits and risks

The benefit of this trial is that we will determine whether pre-emptive administration of morphine provides effective pain relief for acute procedural pain in infants. Not only would effective pain relief make the infants more comfortable during these procedures but it may also improve the physiological stability of the infants in the 24-hour period after the procedure. Furthermore, the provision of effective pain relief may reduce not prevent the long-term structural white matter damage and deficits in cognitive ability that have been directly linked to the number of painful experiences that hospitalised infants receive during their neonatal care ^34^.

There are risks associated with the administration of morphine, which include increased hypotension and respiratory depression ^35, 36^. However, in studies in ventilated infants where approximately double the dose of morphine proposed in this trial was used, there was no difference in blood pressure during the first hour after infusion, the fraction of inspired oxygen during the 12 hours after infusion, or the number of infants with hypotension requiring treatment, between the morphine-treated and control not control-treated infants ^36^. The authors do, however, report a trend towards increased hypotension and ventilator requirements, which they considered is likely to be driven by the administration of the morphine. In a separate retrospective study, where safety of morphine administration in non-intubated infants was investigated, significant differences in hypotension were not observed, although there was a suggestion that increased ventilator support was required when 2 infants were included who had been accidentally overdosed – the difference between the groups was not significant when the overdosed infants were excluded ^35^. While there are side effects associated with the administration of morphine – which will be further investigated as part of this study – the benefit of providing adequate pain relief is likely to outweigh the possible adverse consequences. Given that morphine is commonly administered to hospitalised infants the importance of investigating the efficacy and safety of morphine for procedural pain is clear.

### Justification for trial

ROP screening is a clinically-essential procedure which is known to cause pain and distress in infants, and often results in an extended period of clinical instability ^10, 12^. Numerous pain management strategies have been shown to be ineffective for ROP screening, including the use of sucrose, breast milk and local anaesthetic eye drops ^15– 17^. It is highly beneficial to test whether morphine administration reduces acute pain caused by ROP screening and whether it improves clinical stability during the subsequent 24 hours. While the primary objective of this study is to look at the effect of morphine analgesia on well-validated clinical pain scores calculated 30 seconds after ROP screening, this study also provides an opportunity to gain a mechanistic insight into how morphine alters nociceptive brain and spinal cord activity evoked by an acute noxious heel lance. If a bolus dose of oral morphine is proven to be a safe and effective analgesic for procedural pain that results in improved clinical stability, this would result in a significant change to current clinical practice for ROP screening and, potentially, provide an analgesic which can be used for other acutely painful procedures in infants, such as laser eye surgery.

A single dose of 200 μg/kg oral morphine sulphate (which is double the dose proposed in this study) has been used in a previous study testing analgesic efficacy for ROP screening and the authors reported, in the paper and through personal communication, that there were no adverse effects of morphine administration at this dose ^24^.

Morphine analgesia will be administered in addition to current pain management strategies, which, in the John Radcliffe Hospital, include the use of local anaesthetic eye drops prior to the ROP screening and comfort techniques (such as swaddling) during heel lancing. Other than the administration of the trial medication and some extra physiological monitoring, current clinical practice will not be altered for trial participants.

In this trial we will use a standardised comfort technique. Trial participants will be swaddled using methods that we have successfully implemented in previous electroencephalography (EEG) and Magnetic Resonance Imaging (MRI) studies. We will lay the infant supine on a cotton cloth and cross the infant’s arms over their chest in a relaxed position. The ends of the cloth will then be crossed over the infant’s body and arms, and tucked beneath the opposing side. The swaddling cloth will not cover the infant’s feet, in order to allow access for monitoring leads and blood sampling. The cloth will only restrict gross upper body movements and hold the infant securely and comfortingly in a flexed position.

### Outline of trial proposal

In this study we aim to determine whether a single dose of morphine sulphate (100 μg/kg) administered orally prior to painful clinical procedures provides effective analgesia. The dosage, route of administration and time course have been selected based on previous literature ^24^ and based on recommendations in the British National Formulary for children (BNFc) (2015).

**Figure 1.**
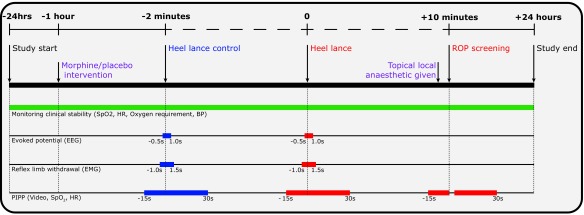
Summary of the experimental protocol. The clinical intervention is defined as the heel lance followed by the ROP screening. All timings are approximate and are in relation to the start of the clinical intervention (i.e. the heel lance).

The primary objective is to determine whether morphine analgesia reduces clinical pain scores following ROP screening. In addition, to provide a better mechanistic understanding of how morphine affects nociceptive brain and spinal cord activity we will investigate the effect of morphine on electrophysiological measures of nociceptive brain and spinal cord activity evoked by a heel lance. Infants will be randomised to receive either morphine or a placebo prior to a clinically required heel lance and ROP screening, which will be performed on the same test occasion. The effect of morphine on clinical pain scores (evoked by the heel lance and ROP screening), nociceptive-specific brain activity and spinal cord activity (evoked by clinical heel lance), clinical stability, and drug safety will be investigated ( Figure 1).

Box 1. **Objectives and Outcome Measures**

**Table:** 

Objectives	Outcome Measures
**Primary Objective** (i) To test whether administration of morphine reduces clinical pain scores (PIPP-R) during the 30 second period after ROP screening compared with a placebo (inactive solution).	**Primary Outcome Measure** (i) PIPP-R score during the 30 second period after ROP screening.
**Co-primary Objective** (ii) To test whether administration of morphine reduces nociceptive-specific brain activity following a clinically-essential heel lance compared with a placebo (inactive solution).	**Co-primary Outcome Measure** (ii) Magnitude of nociceptive-specific brain activity evoked by heel lance.
**Secondary Objectives** (iii) To test whether administration of morphine improves clinical stability in the 6-hour and 24-hour period following the start of the clinical intervention. The clinical intervention is defined as the heel lance followed by ROP screening.	**Secondary Outcome Measures** (iii) Clinical stability in the 6-hour and 24-hour period following the start of the clinical intervention. The clinical intervention is defined as the heel lance followed by ROP screening. (Clinical stability is assessed from pulse oximetry recordings and the need for increased respiratory support.)
(iv) To test whether administration of morphine reduces clinical pain scores (PIPP-R) and reflex withdrawal activity following a clinically essential heel lance compared with a placebo (inactive solution).	(iv) PIPP-R score and amplitude of reflex withdrawal following heel lance.
(v) To test whether administration of morphine is safe by determining whether it results in episodes of respiratory depression or hypotension that require intervention.	(v) Drug safety will be assessed by calculating the number of incidences of apnoea that require intervention using *NeoPuff* or ‘bag and mask’ and the number of incidences of hypotension that require treatment with inotropes in the 24-hour period following the administration of the IMP or placebo.

## Trial design

### Trial description

**Figure 2.**
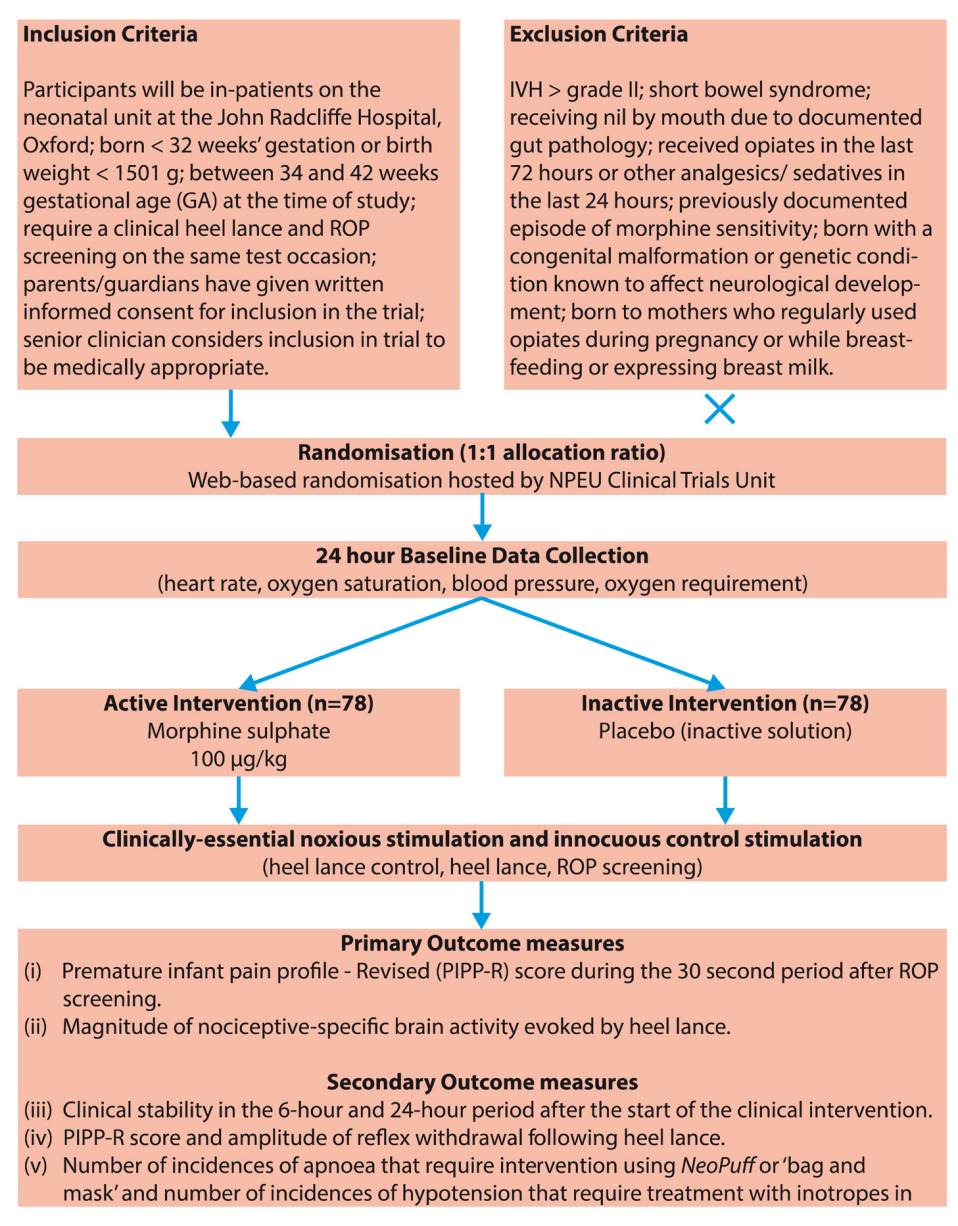
Trial flowchart.

This study is a clinical trial of an investigational medicinal product (IMP). It is a single-centre double-blind randomised placebo-controlled study investigating whether morphine sulphate provides effective analgesia compared with a placebo (inactive solution) for clinically required ROP screening and heel lancing ( Figure 2).

Infants will be recruited during a 33-month period. Infant participants will be studied on a single test occasion while they are in hospital, when they require ROP screening and a clinical heel lance on the same occasion. No extra blood tests or noxious procedures will be performed for the purpose of the study. Participants will be included in the study for a 48-hour period. This will be 24-hours before and after the start of the clinical intervention. The clinical intervention is defined as the heel lance followed by the ROP screening. The start of the clinical intervention is therefore defined as the time when the heel lance is performed.

## Participant identification

### Trial participants and study feasibility

6500 infants are born at the John Radcliffe Hospital each year and 700 are admitted to the neonatal unit. ROP screening is performed on approximately 100 infants per year and on average these infants will receive two ROP exams while they are in-patients. The majority of infants will require routine blood tests prior to the ROP screening and, based on previous studies we expect a recruitment consent success rate of approximately 60%.

Each week the Ophthalmologists perform up to 10 ROP screening examinations in the neonatal unit. Of these we expect that approximately 3 examinations would be conducted on infants who are eligible for inclusion in the trial. In our previous study ^32^, which used a similar design (with an active and placebo arm) the parental consent rate was 66% (62 out of 94 infants were successfully recruited). To keep to our proposed recruitment schedule (156 infants in 33 months), on average, we need to recruit just over 1 baby per week. This equates to a parental consent rate of approximately 30%, which we consider to be realistic and highly achievable.

We plan to study 1–2 infants per week and intend to study 156 infants in total, suggesting that it is feasible that the data is collected for this trial within 2 years and 9 months. We expect to approach approximately 400 parents and will not study any individual baby more than once.

### Inclusion criteria

• Participants will be in-patients on the neonatal unit at the John Radcliffe Hospital, Oxford.
• Infants born less than 32 weeks’ gestation or birth weight less than 1501 g.
• At the time of study, infants will be between 34 and 42 weeks gestational age (GA) and will be studied if they require a clinical heel lance and ROP screening on the same test occasion. We will study infants during a single test occasion when they are greater than or equal to 34 weeks’ gestation.
• Infants for whom parents/guardians have given written informed consent for inclusion in the trial.
• Senior clinician considers inclusion in trial to be medically appropriate.

### Exclusion criteria

• intraventricular haemorrhage (IVH) > grade II
• short bowel syndrome
• receiving nil by mouth due to documented gut pathology
• received opiates in the last 72 hours
• received other analgesics or sedatives in the last 24 hours
• previously documented episode of morphine sensitivity
• congenital malformation or genetic condition known to affect neurological development
• born to mothers who regularly used opiates during pregnancy or while breastfeeding or expressing breast milk.

## Trial procedures

### Recruitment

The clinical team will identify infants who are eligible for inclusion in the study shortly after birth and the infant’s parents will be informed about the study. Parents who are interested in the study will be given the opportunity to ask questions and will be given a Parent Information Leaflet (PIL). Once an ROP screening appointment has been confirmed in infants between 34 and 42 weeks gestation (which will be at least two weeks after they have been born), parents will be asked by members of the research team if they would like to give informed consent for their infant to take part in the study.

### Informed consent

Written consent will be sought from the parents after they have been given a full verbal and written explanation of the trial. Written explanation will be given via the Parent Information Leaflet (PIL). Parents who do not speak English will only be approached if an adult interpreter is available. Relatives will not interpret.

Written informed parental consent will be obtained by means of dated parental signature and the signature of the person who obtained informed consent; this will be the Principal Investigator (PI) or healthcare/research professional with delegated authority. A copy of the signed informed consent form (ICF) will be given to the parent(s). A further copy will be retained in the infant’s medical notes, a copy will be retained by the PI, and the original will be stored with the study data.

### Enrolment

After informed consent has been obtained, the information will be entered onto the randomisation website. Infants will be considered enrolled into the Poppi trial once they have been given a study number.

### Remuneration

Neither trial participants nor their parents will be given financial or material incentive or compensation to take part in this trial.

### Randomisation

Randomisation of participants to receive either morphine sulphate or placebo (inactive solution) will be managed via a secure web-based randomisation facility hosted by the National Perinatal Epidemiology Unit Clinical Trials Unit (NPEU CTU) at The University of Oxford. Participants will have a roughly equal chance of receiving morphine sulphate or placebo (inactive solution). The randomisation program will use a minimisation algorithm to ensure approximate balance between the groups with respect to gestational age at the time of study, gestational age at birth, number of days on a ventilator, presence of gastric tube at time of study, number of days since morphine has been given, intra-uterine growth restriction and previous surgery. The users of the system will have no insight into the next allocation. Balance between the groups for each of the minimisation variables will be reviewed by the Data Monitoring Committee (DMC) and reported in trial publications.

Participants will be randomised shortly before the 48-hour period of clinical stability monitoring commences. If any of the exclusion criteria manifest after consent but prior to randomisation, participants will not be randomised and therefore not included in the trial. If exclusion criteria manifest in the first 24 hours of clinical stability monitoring the investigational medicinal product (IMP) or placebo will not be administered and the infant withdrawn from the trial. In this case the infant will still be considered a trial participant.

A Senior Trials Programmer at the NPEU CTU will write the randomisation program and hold the code. In the event of an emergency, a participant may be unblinded by logging in to the randomisation website using a single-use access code provided in a sealed envelope. The reason for unblinding must be recorded. Clinicians carrying out emergency unblinding must be satisfied that it is a genuine emergency and that knowledge of the treatment allocation (either morphine or placebo) is needed to guide the appropriate clinical management of the participant. In some cases this may be achieved without unblinding by treating the participant as if they have received morphine sulphate solution.

### Blinding

This will be a blinded study. Each individual IMP pack will contain 10ml amber glass bottle with tamper evident cap containing either morphine sulphate or placebo (inactive solution) and a sterile oral/enteral 3ml syringe. They will be labelled with a randomisation code and will be indistinguishable by colour, smell and flow during administration. The packaging for both morphine sulphate and placebo will be identical. Once an infant is recruited into the trial, they will receive a single dose of morphine or placebo. The IMP packs will be stored in the study-specific drug cabinet in the neonatal unit.

When the study has officially ended and the data analysis is complete the randomisation code will be released to the primary researchers and statistician so that the treatment groups can be correctly identified. Thus, throughout the study all of the primary researchers, outcome assessors, parents and infants will be blinded to the identities of the solutions.

### Trial design summary

Each recruited infant will participate in the trial for 48 hours, and will not require further follow-up. Once recruited, we will establish when the ROP screening is to be conducted and the studies will be timed to fit in with the Ophthalmology schedule. The Consultant Ophthalmologist is fully informed about this study and has agreed to liaise with the research team regarding timing of the ROP screening. Infants will be randomised to receive a single dose of 100 μg/kg morphine sulphate or placebo, administered orally or via a gastric tube.

The IMP will be provided in individual 10ml amber glass bottles containing morphine sulphate (or inactive solution) at a concentration of 200 μg/ml and will be administered orally or via a gastric tube using a syringe 60 minutes before the heel lance is undertaken. The volume of solution administered to each infant will be calculated based on the infant’s working weight at the time of the study.

60 minutes before the heel lance (and after randomisation) infants will be administered either morphine sulphate or a placebo. Once the drug has been administered the research team will set up the electroencephalography (EEG), electromyography (EMG) and video monitoring. When the drug has reached maximum efficacy (which is approximately 60 minutes after administration) a clinical heel lance and heel lance control stimuli (where the lancet is rotated such that on release the blade does not touch the foot) will be performed. During each procedure nociceptive-specific brain activity (measured using EEG), reflex withdrawal activity (measured using EMG on the biceps femoris of each leg), physiological activity (including oxygen saturation and heart rate) and facial expression change will be recorded.

After the heel lance has been completed, routine ROP screening will be carried out by an Ophthalmologist or appropriately trained Ophthalmology Trainee. The time taken for the screening to be performed will be recorded.

Once the ROP screening is complete, pulse oximetry, oxygen requirement and blood pressure will continue to be monitored and recorded by our data logging equipment for 24 hours after the start of the clinical intervention (heel lance followed by ROP screening). Clinical stability of the infants will be assessed throughout the 48-hour trial period. These measures will be calculated from pulse oximetry recordings and requirement for respiratory support. Pulse oximetry data will be monitored and downloaded to our data logging equipment for 24 hours before and 24 hours after the start of the clinical intervention (heel lance followed by ROP screening). Throughout the 48-hour trial period, blood pressure will be monitored 6 hourly and changes in respiratory support (including type of respiratory support modality and oxygen requirement) will be recorded.

### Heel lance procedure

Infants will only be given a heel lance during routine investigations when blood samples are clinically required. Given the frequency with which blood samples are required in premature infants, the heel lance can be timed to occur immediately before the ROP screening. The heel lancet will be linked electronically to the recording equipment, using methods that have been used in previous studies ^30– 32^. This methodology provides an opportunity to record the precise timing of when the heel lance occurs without interfering with clinical practice.

### Retinopathy of prematurity (ROP) screening

All infants born at less than 32 weeks gestation or with a birth weight less than 1501 g are screened for ROP (UK Retinopathy of Prematurity Guidelines, 2008). The decision to conduct ROP screening will be independent of the trial. National and local policy guidelines will be followed to determine when screening will be performed (UK Retinopathy of Prematurity Guidelines, 2008). An Ophthalmologist or suitably experienced Ophthalmology Trainee will carry out screening as per standard practice. Both pupils will be dilated with mydriatic eye drops (Tropicamide 1% and Phenylephrine 2.5%) approximately 1 hour prior to screening. At the time of screening, an assistant will support the infant’s head. Topical local anaesthetic (proxymetacaine 0.5%) instillation will be followed 10 seconds later by insertion of an eyelid speculum, which will be used to hold the eyelid open during the exam. The eye will be intermittently lubricated using sterile saline drops. A Flynn style indenter will be used to stabilise the eye allowing for a standardised intensity light beam to enter the eye through a condensing lens as part of the binocular ophthalmoscopic fundus examination. The right eye will be examined first followed by removal of the speculum and insertion into the left eye to repeat the process.

### Recording techniques

We will use a range of techniques (listed in Table 1) to characterise the responses to noxious stimulation. Some of these measures will not be an additional burden for the infants as they are continuously recorded as part of their routine clinical care (i.e. pulse oximetry).

**Table 1.** Techniques used to characterise infant behaviour and physiology

Procedures	Recording Methods	Measurement
1	Electroencephalography (EEG)	Nociceptive-specific brain activity
2	Electromyography (EMG)	Reflex withdrawal
3 *	Pulse Oximetry	Heart rate
4 *	Pulse Oximetry	Oxygen saturation
5	Video recording	Facial expression change
6 *	Blood pressure monitor	Blood pressure
7 *	Oxygen flow meter (if required)	Oxygen requirement

Note: (*) starred procedures are monitored as part of routine clinical care

***Electroencephalography (EEG).*** Electrophysiological activity will be acquired with the SynAmps RT 64-channel headbox and amplifiers (Compumedics Neuroscan), with a bandwidth from DC - 400 Hz and a sampling rate of 2 kHz. CURRYscan7 neuroimaging suite (Compumedics Neuroscan) will be used to record the activity. All equipment will conform to the electrical safety standard for medical devices, IEC 60601–1. Eight EEG recording electrodes will be positioned on the scalp at Cz, CPz, C3, C4, FCz, T3, T4 and Oz according to the modified international 10–20 System. Reference and ground electrodes will be placed at Fz and the forehead respectively. EEG conductive paste will be used to optimise contact with the scalp. All impedances will be reduced to approximately 5 kΩ by rubbing the skin with EEG preparation gel prior to electrode placement. An ECG electrode will be placed on the left clavicle and a movement transducer will be placed on the abdomen to record respiration.

***Electromyography (EMG).*** Bipolar EMG electrodes (Ambu Neuroline 700 solid gel surface electrodes) will be placed on the biceps femoris of each leg to measure reflex withdrawal. EMG will be recorded before and after the heel lance control and the heel lance stimuli.

***Clinical pain scores.*** Clinical pain scores will be calculated using the validated Premature Infant Pain Profile - Revised (PIPP-R). PIPP-R is a composite measure encompassing behavioural, physiological and contextual indicators involved in the pain response. These include gestational age, behavioural state, heart rate, oxygen saturation, and duration of brow bulge, eye squeeze, and nasolabial furrow. Each indicator in the PIPP-R is rated on a 4-point scale (0, 1, 2, and 3); these are summed together to produce a maximum possible score of 21. In the recently revised PIPP-R, the scores for the contextual indicators (gestational age and behavioural state) are only included if a non-zero score is recorded for either the physiological or behavioural variables ^26^.

Videos of facial expression will be recorded throughout the procedures and scored offline from single frames using the PIPP-R facial coding system. Changes in heart rate and oxygen saturation will be downloaded from the pulse oximeter and used to calculate the PIPP-R score.

Heart rate, oxygen saturation and facial expression will be recorded in the 15-second period before and 30-second period after the heel lance and heel lance control. These measures will also be recorded in the 15-second period before ROP screening and in a 30-second period after ROP screening has been completed ^25, 26^.

***Pulse oximetry.*** Heart rate and oxygen saturation will be measured throughout the 48-hour study period and downloaded to a computer. These data will be used to calculate the clinical pain scores following the heel lance and ROP screening and to assess clinical stability during the 6-hour and 24-hour after the start of the clinical intervention. (The clinical intervention is defined as the heel lance followed by ROP screening.) Data will be analysed offline and the number of clinically relevant episodes will be calculated based on the following definitions:

• an episode of bradycardia will be defined as a pulse rate < 100 beats/min for at least 15 s
• an episode of tachycardia will be defined as a pulse rate > 200 beats/min for at least 15 s
• an episode of desaturation will be defined as oxygen saturation < 80% for at least 10 s
• an episode of apnoea will be defined as the cessation of respiratory air flow for at least 20 s
Episodes of artefact (i.e. infant handling or essential clinical procedures) will be documented throughout the recordings.

***Blood pressure monitoring.*** Blood pressure will be monitored 6-hourly during the 24-hour period before and after the start of the clinical intervention. (The clinical intervention is defined as the heel lance followed by ROP screening.) This will be measured using a standard blood pressure cuff.

***Increased respiratory support.*** Any change in respiratory support modality or a significant change in oxygen requirement will be documented during the 24-hour period before and after the start of the clinical intervention. (The clinical intervention is defined as the heel lance followed by ROP screening.)

Increased respiratory support will be defined as a significant increase in oxygen requirement or an increase in ‘respiratory support modality’.

**Respiratory support modality** is classified on a graded scale from 1–4, according to the following definitions.

Grade 1: self-ventilating in air
Grade 2: low flow (0.01–0.35 litres/minute; 100% oxygen)
Grade 3: high flow (1–8 litres/minute) or continuous positive airway pressure (CPAP) or duoPAP (21–100% oxygen)
Grade 4: ventilator (21–100% oxygen)

If an infant has a change in ‘ **respiratory support modality**’ that results in an increase in grade, this will count as an increase in **respiratory support**. 

A **significant increase in** **oxygen requirement** is defined as an increase in oxygen supply by more than 10%, a flow rate change of more than 1 litre/minute (if receiving high flow therapy) or a flow rate change of more than 0.04 litres/minute (if receiving low flow oxygen).

***Measures of clinical stability.*** Clinical stability will be assessed based on the following measures (calculated in the 6-hour and 24-hour period before and after the start of the clinical intervention): number of episodes of bradycardia; number of episodes of tachycardia; number of episodes of desaturation; number of episodes of apnoea; and number of infants that required an increase in respiratory support.

### Outcome measures

***Premature Infant Pain Profile - Revised (PIPP-R) scores.*** Pain scores will be calculated using the Premature Infant Pain Profile-Revised (PIPP-R) scoring system ^26^. PIPP-R scores will be calculated on three occasions: (i) during the heel lance control procedure; (ii) during the heel lance procedure; and (iii) during the 30 second period after the ROP screening (it is not possible to accurately calculate PIPP-R scores during ROP screening because the eyes are held open by a speculum). An interim analysis will be conducted once data has been collected from 50% of the trial participants. 20% of PIPP-R scores will be re-calculated by two investigators to ensure inter-rater and intra-rater reliability. Inter-rater and intra-rater reliability will also be calculated in 20% of trial participants once all data has been collected.

***Nociceptive-specific brain activity.*** Nociceptive-specific brain activity evoked by a clinical heel lance has been well characterised in previous studies ^30– 32^. In this study we will investigate whether morphine administered prior to a clinically-required heel lance reduces the nociceptive-specific brain activity compared to a placebo (inactive solution). A mathematical template based on the Principal Component (PC) that reflects the nociceptive-specific activity in infants has been defined in an independent data set. The nociceptive-specific template will be projected onto the EEG data recorded in the 1000 ms period following each heel lance and heel lance control stimulus and the relative weight of the component calculated for each infant. A greater weight indicates a stronger noxious-specific evoked response. While the brain activity characterised here is directly related to nociceptive input, it does not reflect all nociceptive activity that takes place across the brain or all aspects of the pain experience. The response to the heel lance control stimulus is being recorded so that we can confirm that the brain activity evoked by the heel lance is noxious. This forms an important data quality control check ^30^.

***Reflex withdrawal.*** The reflex withdrawal response will be characterised and the effect of treatment considered. The EMG data will be high pass filtered at 10 Hz and low pass filtered at 500 Hz. The data will be baseline corrected to the pre-stimulus mean and the root mean square (RMS) of the signal calculated in 250 ms windows ^37^. The average root mean squared (RMS) value will be calculated in the 500 ms pre-stimulation and in the 1000 ms post-stimulation ^32, 37^.

***Clinical stability.*** Clinical stability will be assessed based on 5 clinical stability measures calculated in the 6-hour and 24-hour period before and after the start of the clinical intervention. (The clinical intervention is defined as the heel lance followed by ROP screening.) The following outcome measures will be calculated: number of episodes of bradycardia; number of episodes of tachycardia; number of episodes of desaturation; number of episodes of apnoea; and number of infants that required an increase in respiratory support.

***Safety of administering a single bolus dose of oral morphine.*** The final objective of this study is to establish whether the administration of this dose of oral morphine is safe.

This will be established by calculating the number of incidences of apnoea that require intervention using *NeoPuff* or ‘bag and mask’ and the number of incidences of hypotension that require treatment with inotropes in the 24-hour period following the administration of the IMP or placebo. This information will contribute considerably to the safety profile of oral morphine in neonatal patients.

### Procedure for unblinding

In the event of an emergency, a participant may be unblinded by the clinician at site by logging in to the randomisation website using a single-use access code provided in a sealed envelope. The reason for unblinding must be recorded. Clinicians carrying out emergency unblinding must be satisfied that it is a genuine emergency and that knowledge of the treatment allocation (either morphine or placebo) is needed to guide the appropriate clinical management of the participant. In some cases this may be achieved without unblinding by treating the participant as if they have received morphine sulphate solution.

As it is best practice to not unblind participants until any follow-up is completed, all other requests for unblinding must be made in writing to the NPEU CTU, who along with Chief Investigator and Principal Investigator will consider the request.

### Withdrawal from the intervention

Parent(s) may withdraw their infant from the intervention at any time and they are not obliged to give a reason. If parents choose to withdraw their child after the IMP or placebo (inactive solution) has been administered, they will be asked whether data already collected may be retained and used for the purposes of the trial. Parents will be made aware that this decision has no impact on any aspects of their infant’s continuing care.

The attending clinician may withdraw the infant from treatment if they consider this to be in the best interest of the infant’s health and well-being. If any of the exclusion criteria manifest in the 24 hours of clinical stability monitoring prior to the administration of the IMP or placebo then the infant will be withdrawn from the trial.

### Definition of end of trial

The end of the trial will be defined as the date when the trial database is locked. An end of trial declaration will be made to MHRA and the approving REC.

## Investigational Medicinal Product (IMP)

### Description of IMP and placebo

The IMP is morphine sulphate prepared in a carrier solution so that it is suitable for oral administration in infants. The placebo contains the carrier solution without the morphine sulphate. The IMP and placebo must be stored at a temperature of 25°C or below.

Individual identical 10ml glass amber bottles with tamper evident cap containing either morphine sulphate or placebo (inactive solution) will be labelled with a pack ID and will be indistinguishable by colour, smell and flow during administration. The secondary packaging will contain a single use sterile oral/enteral 3ml syringe and will be labelled with a pack ID. The morphine sulphate will be supplied at a concentration of 200 μg/ml, in a dose of 100 μg/kg. This dose has been selected based on recommendations in the BNFc (British National Formulary for children (2015)).

### Preparation

The IMP and placebo (inactive solution) will be manufactured by Stockport Pharmaceuticals Production Unit in accordance with Good Manufacturing Practice (GMP).

### Distribution

The IMP will be delivered to the Hospital Pharmacy at the John Radcliffe Hospital.

### Storage of IMP and placebo (inactive solution)

The trial medication will be stored in the study-specific locked drug cabinet in the Neonatal Unit.

### Accountability

The drugs will be allocated by the central randomisation system and will be recorded by NPEU CTU and the Neonatal Unit Pharmacist. A record of the individual administrations and drug accountability will be kept.

Studies will be conducted on the Neonatal Unit where specialist doctors and nurses are present at all times. Resuscitation equipment is available if necessary, including naloxone (an antagonist for morphine).

### Destruction

The volume of the IMP or placebo to be discarded will be disposed of as per local trust policy. It will be rendered irretrievable by emptying the precise calculated volume for destruction into a sharps bin. This process will be witnessed by a second qualified clinical staff member. The volume discarded will be clearly documented by the clinician undertaking the destruction and the witness. The syringe, once emptied during IMP administration, will then also be discarded in the sharps bin. In the event that the IMP reaches its expiry date before use, it will be retrieved by pharmacy and destroyed as per trust protocol.

### Concomitant medication

Current clinical protocols regarding drug administration on the Neonatal Unit will not be altered by this study (apart from the additional trial medication). At present infants in the Neonatal Unit where the study is being conducted do not receive pain medication prior to heel lancing, but comfort measures such as swaddling are provided. It is therefore acceptable that the control group receive a placebo (inactive solution) with no medicinal effects. After the heel lance, and approximately 10 seconds before the ROP screen, all infants will receive 0.5% proxymetacaine local anaesthetic in each eye, following standard clinical practice. In addition, approximately 1 hour prior to the ROP screen, all infants will also receive mydriatic eye drops to evoke pupil dilation. The infants may be receiving medication for other conditions and the attending clinician will confirm that participation in the trial is not contraindicated.

## Safety reporting

Box 2. **Safety Reporting Definitions**

**Table:** 

Adverse Event (AE)	Any untoward medical occurrence in a participant to whom a medicinal product has been administered, including occurrences which are not necessarily caused by or related to that product.
Adverse Reaction (AR)	An untoward and unintended response in a participant to an investigational medicinal product which is related to any dose administered to that participant. The phrase "response to an investigational medicinal product" means that a causal relationship between a trial medication and an AE is at least a reasonable possibility, i.e. the relationship cannot be ruled out. All cases judged by either the reporting medically qualified professional or the Sponsor as having a reasonable suspected causal relationship to the trial medication qualify as adverse reactions.
Serious Adverse Event (SAE)	A serious adverse event is any untoward medical occurrence that: • results in death • is life-threatening • requires inpatient hospitalisation or prolongation of existing hospitalisation • results in persistent or significant disability/incapacity • consists of a congenital anomaly or birth defect. Other ‘important medical events’ may also be considered serious if they jeopardise the participant or require an intervention to prevent one of the above consequences. NOTE: The term "life-threatening" in the definition of "serious" refers to an event in which the participant was at risk of death at the time of the event; it does not refer to an event which hypothetically might have caused death if it were more severe.
Serious Adverse Reaction (SAR)	An adverse event that is both serious and, in the opinion of the reporting Investigator, believed with reasonable probability to be due to one of the trial treatments, based on the information provided.
Suspected Unexpected Serious Adverse Reaction (SUSAR)	A serious adverse reaction, the nature and severity of which is not consistent with the information about the medicinal product in question set out: • in the case of a product with a marketing authorisation, in the summary of product characteristics (SmPC) for that product • in the case of any other investigational medicinal product, in the investigator’s brochure (IB) relating to the trial in question.

NB: to avoid confusion or misunderstanding of the difference between the terms “serious” and “severe”, the following note of clarification is provided: “Severe” is often used to describe intensity of a specific event, which may be of relatively minor medical significance. “Seriousness” is the regulatory definition supplied above.

### Causality

The relationship of each adverse event to the trial medication must be determined by a medically qualified individual according to the following definitions:

• **Unrelated** – where an event is not considered to be related to the IMP.
• **Possibly** – although a relationship to the IMP cannot be completely ruled out, the nature of the event, the underlying disease, concomitant medication or temporal relationship make other explanations possible.
• **Probably** – the temporal relationship and absence of a more likely explanation suggest the event could be related to the IMP.
• **Definitely** – the known effects of the IMP, its therapeutic class or based on challenge testing suggest that the IMP is the most likely cause.
All AEs (SAEs) labelled possibly, probably or definitely will be considered as related to the IMP.

### Procedures for recording adverse events

The following are adverse events that could be reasonably foreseen to occur in this population of infants during the course of this trial and do not require recording.

• Suspected sepsis (requiring up to 36 hours antibiotics)
• Anaemia
• Minor changes in oxygen requirement (i.e. an increase in oxygen supply by less than 10%, a flow rate change of less than 1 litre/minute (high flow machine) or a flow rate change of less than 0.04 litres/minute (low flow machine)
• Electrolyte disturbances

All other AEs occurring during the 24 hour period following the administration of the IMP or placebo that are observed by the Investigator, will be recorded on the Case Report Form (CRF), whether or not attributed to trial medication.

The following information will be recorded: description, time and date of onset, severity, assessment of relatedness to trial medication, other suspect drug or device and action taken. Follow-up information should be provided as necessary.

The severity of events will be assessed on the following scale: 1 = mild, 2 = moderate, 3 = severe.

AEs considered related to the trial medication as judged by a medically qualified investigator or the Sponsor will be followed up either until resolution, or the event is considered stable.

It will be left to the Investigator’s clinical judgment to decide whether or not an AE is of sufficient severity to require intervention and or removal from the trial. If this occurs, the participant will be given medical supervision until symptoms cease, or the condition becomes stable.

### Reporting procedures for serious adverse events

***Foreseeable serious adverse events.*** The following are serious adverse events that could be reasonably foreseen to occur in this population of infants during the course of this trial. They do not require expedited reporting by the trial centre as SAEs.

• Death (unless unforeseen)
• Necrotising enterocolitis (NEC) or focal intestinal perforation
• Intracranial abnormality (haemorrhage, parenchymal infarction, or focal white matter damage) on cranial ultrasound scan or other imaging
• Microbiologically-confirmed or clinically suspected late-onset invasive infection
• Retinopathy of prematurity
• Patent ductus arteriosus (requiring treatment)
• Congenital abnormalities

***Reporting procedures for serious adverse events.*** Safety data as described in this section will be collected for 24 hours after the IMP or placebo is administered. All foreseeable SAEs will be recorded on the eCRF. SAEs that are not included in the list of foreseeable SAEs will be reported to NPEU CTU immediately, but at least within 24 hours of the research site becoming aware of the event. SAEs can be reported in one of the following ways:

i) using the Clinical Database OpenClinica©, only staff with access to OpenClinica© may report SAEs in this way, site staff will be required to print off the OpenClinica© SAE form and obtain the information and signature of the Study Clinician carrying out the causality assessment. The completed signed SAE form must be emailed or faxed to NPEU CTU. NPEU CTU staff will automatically be informed via email of any SAE’s reported electronically.
ii) by completing an SAE form which is emailed or faxed to NPEU CTU. Paper copies will be available with the trial documentation to enable anyone to report an SAE.

NPEU CTU will ensure that the SAEs are assessed by the Chief Investigator (CI) or safety delegate and that review of the SAEs are timely, taking into account the reporting time for a potential SUSAR. If any additional information regarding the SAEs becomes available this will be detailed on a new SAE form and also emailed or faxed to NPEU CTU or reported electronically using OpenClinica©. If reporting electronically, the site must complete the information in OpenClinica© and arrange for it to be printed and signed by the Study Clinician making the causality assessment. Guidance for the research site is provided on the paper SAE reporting form and contained within trial guidance documentation. All SAEs will be reviewed by the DMC after every 25 patients have been randomised and safety data collected (i.e. n=25, 50, 75, 100 and 125). The CI will inform all investigators concerned of relevant information that could adversely affect the safety of participants.

***Expectedness.*** Expectedness will be determined according to the Summary of Product Characteristics for Oramorph Oral Solution and will be assessed by a medically qualified investigator or suitably qualified delegate.

***Suspected unexpected serious adverse reactions (SUSAR) reporting.*** All SUSARs will be reported by the CI or delegate to the relevant Competent Authority and to the REC and other parties as applicable. For fatal and life-threatening SUSARs, this will be done no later than 7 calendar days after the Sponsor or delegate is first aware of the reaction. Any additional relevant information will be reported within 8 calendar days of the initial report. All other SUSARs will be reported within 15 calendar days.

Treatment codes will be unblinded for specific participants.

The Sponsor will inform all Principal Investigators of any SUSARs for the relevant IMP for all studies they are sponsoring, whether or not the event occurred in the current trial.

### Development Safety Update Reports (DSURs)

The CI will submit (in addition to the expedited reporting above) DSURs once a year throughout the clinical trial, or on request, to the Competent Authority (MHRA in the UK), Ethics Committee, Host NHS Trust and Sponsor.

## Statistics and Analysis

### Power calculation

The primary objective of this study is to determine whether morphine administration reduces clinical pain scores (PIPP-R) during the 30 second period after ROP screening in the infants administered morphine compared with infants administered a placebo (inactive solution). The co-primary objective is to determine whether morphine administration reduces nociceptive-specific brain activity in response to a heel lance, compared with administration of a placebo. A 2-point reduction in PIPP scores is considered to be a clinically-meaningful reduction in pain ^38^ and scores below 7 are considered to reflect minimal pain ^25^. Three studies have used PIPP scores to assess analgesic efficacy following ROP screening ^39– 41^. Using the most conservative data, where the mean PIPP score post ROP screening was 8.3 (standard deviation [SD] 3.5), we would require 66 infants per group to observe a 2-point reduction in PIPP-R scores, with a power of 90% at a two-sided significance level of 0.05.

We also considered the sample size required to see a significant difference between the groups in the co-primary outcome measure (i.e. the nociceptive-specific brain activity evoked by a heel lance). In previous analysis ^33^ the nociceptive-specific brain activity evoked by a heel lance had a mean amplitude of 0.2 (SD 0.14). In this study the amplitude of the nociceptive-specific brain activity will be calculated and compared between the two groups. Studies in adults with chronic pain indicate that morphine treatment attenuates the amplitude of laser-evoked potentials by 33.1% ^42^. Furthermore, adults administered Tramadol (an opioid treatment) show up to a 50% reduction in the amplitude of laser-evoked potentials ^43^. This reduction in nociceptive brain activity is coupled with the verbal report that tramadol is providing effective pain relief. Tramadol is a widely used as an analgesic in adults and trials have shown that it effectively reduces pain in patients with neuropathic pain ^44, 45^.

In this study we will assume that a 40% reduction in the magnitude of the nociceptive-specific brain activity represents a clinically meaningful reduction in brain activity, which in adults would be concomitant with a significant reduction in verbally reported pain scores. A 40% reduction would lead to the noxious components having a post-treatment amplitude of approximately 0.12 (SD 0.14) down from a mean of 0.20. Coincidentally, we would require 66 infants per group for a power of 90% at a two-sided significance level of 5%. Allowing for a loss to follow-up rate of approximately 10% (i.e. due to technical difficulties during physiological monitoring), a total of 148 infants will need to be included in the study.

We anticipate the proportion of twins eligible for the trial to be around 25% based on past studies (e.g. NPEU-run BOOST-II UK, I2S2, PiPS and SIFT trials). There is evidence that the correlation in pain outcomes between twins is 0.5 ^46^. Hence the effect of clustering (technically the design effect) is calculated to be 1.06. This will inflate the total sample size required (90% power, 5% two-sided significance level, 10% loss to follow-up and accounting for multiple births) from 148 to 156 (78 per group).

### Significance levels

For the analysis of the primary outcome measure and co-primary outcome measure, a P-value of 0.05 (two-sided 5% significance level) will be used to indicate statistical significance. We will address the multiplicity issue by using Hochberg’s procedures for multiple testing to control the family-wise error rate ^47^. Therefore, we will reject the null hypothesis for both outcomes if P<0.05 for both outcomes. If P>0.05 for one of the outcomes then the other outcome will be tested at 2.5% significance level. This method is less stringent than the Bonferroni adjustment while preserving the original power of the study.

Comparisons of all other outcomes will be reported in full for completeness and transparency. For all other analyses, a P-value of 0.01 (two-sided 1% significance level) will be used to indicate statistical significance, in order to take account of the number of comparisons. Two-sided statistical tests and corresponding P-values will be presented throughout; however for the purposes of interpretation of results, confidence intervals will dominate, rather than P-values.

### Missing data

Missing data may occur in our trial. For example, this could be due to equipment failure, artefacts within the EEG recording, or the infant being withdrawn if morphine administration is deemed clinically inappropriate after a participant has been randomised. If missing data does exist we expect it will occur at random, and so the collected data will be representative of the population. To account for missing data we have inflated our sample size by 10%. The analysis will be conducted using the available data.

### Analysis

***Primary and co-primary outcome measures analysis.***

***PIPP-R score during the 30 second period after ROP screening*** PIPP-R scores (during the 30 second period after ROP screening) in the morphine and placebo groups will be compared using linear regression to estimate the mean difference with 95% confidence interval. If the PIPP-R scores are skewed, we will present the median and interquartile range (or entire range, whichever is appropriate) for each group, and estimate the median difference between groups with corresponding 95% confidence interval.

***Nociceptive-specific brain activity*** The projected weight of the template for the morphine and placebo groups will be compared using linear regression, depending on the mean and variance of the data. If appropriate, we will present the mean and standard deviation for each group and estimate the mean difference plus 95% confidence intervals. If the outcome data are skewed, we will present the median and interquartile range (or entire range, whichever is appropriate) for each group, and estimate the median difference between groups with corresponding 95% confidence interval.

***Secondary outcome measures analysis.***

***PIPP-R score following heel lance*** PIPP-R scores following heel lance in the morphine and placebo groups will be compared using linear regression to estimate the mean difference with 99% confidence interval. If the PIPP-R scores are skewed, we will present the median and interquartile range (or entire range, whichever is appropriate) for each group, and estimate the median difference between groups with corresponding 99% confidence interval.

***Reflex withdrawal*** The average RMS activity in the 1000 ms post the heel lance for the morphine and placebo groups will be compared using linear regression, depending on the mean and variance of the data. If appropriate, we will present the mean and standard deviation for each group and estimate the mean difference plus 99% confidence interval. If the outcome data are skewed, we will present the median and interquartile range (or entire range, whichever is appropriate) for each group, and estimate the median difference between groups with corresponding 99% confidence interval.

***Clinical stability analysis*** The number of episodes of bradycardia, tachycardia, desaturation, and apnoea; and the number of infants that required an increase in respiratory support that occur in the 24-hour period after the start of the clinical intervention will be compared between the morphine and placebo groups. Depending on the distribution of these counts, either Poisson or linear regression will be used for these analyses to calculate the effect estimate plus 99% confidence interval. If appropriate, adjustment for the 24 hour baseline period will be made. Alternatively, we will present the median and interquartile range (or entire range, whichever is appropriate) for each group and estimate the median difference between groups with corresponding 99% confidence interval. In the event of occurrences being very infrequent or the outcome of interest being the occurrence of increased respiratory support, logistic regression may be used as an alternative.

***Safety analysis*** Drug safety will be assessed by measuring the number of occurrences of apnoea that require intervention via *NeoPuff* or ‘bag and mask’ and the number of episodes of hypotension that require treatment with inotropes in the 24-hour period after drug administration. The number of occurrences will be compared between the morphine and placebo groups. Depending on the distribution of these counts, either Poisson or linear regression will be used for these analyses to calculate the effect estimate plus 99% confidence interval. Alternatively, we will present the median and interquartile range (or entire range, whichever is appropriate) for each group and estimate the median difference between groups with corresponding 99% confidence interval. In the event of occurrences being very infrequent, logistic regression may be used as an alternative.

We currently use Stata/SE 13.1 for Windows for statistical analysis.

## Data Management

### Source data

Source documents are where data are first recorded, and from which participants’ case report form (CRF) data are obtained. These include, but are not limited to, hospital records (from which medical history and previous and concurrent medication may be summarised into the CRF), clinical and office charts, laboratory and pharmacy records, diaries, video footage acquired for the purpose of the trial, physiological data (e.g. EEG recordings, EMG recordings and other physiological data) and correspondence.

CRF entries will be considered source data if the CRF is the site of the original recording (e.g. there is no other written or electronic record of data). All documents will be stored safely in confidential conditions.

### Access to data

Direct access will be granted to the research team, authorised representatives from the Sponsor, host institution and the regulatory authorities to permit trial-related monitoring, audits and inspections.

### Data handling and record keeping

The CI will take overall responsibility for ensuring that each participant’s information remains confidential. All records will be stored securely and kept in strict confidence in compliance with the Data Protection Act. Minimal personal data will be collected and stored at NPEU CTU. Data collected will be stored in an electronic database in which the participant will be identified by a trial specific number. The infant’s name and any other identifying details will be stored separately and linked by the trial number. This information will be stored for a period of no less than 25 years in order to follow up health related issues that may become relevant in the future. After the trial has been completed and the reports published, the data will be archived in a secure physical or electronic location with controlled access.

Storage will be on a restricted area of a file server. The server is in a secure location and access is restricted to a few named individuals. Offices where data are stored are located in a secure area via an electronic tag and individual rooms are kept locked when unoccupied.

Data will be processed on a workstation by authorised staff. The computer workstations access the network via a login name and password (changed regularly). No data are stored on personal workstations. Backing up is done automatically overnight to an offsite storage area. The location of the backup computer is in a separate department.

## Quality Assurance Procedures

The trial will be conducted in accordance with the current approved protocol, Good Clinical Practice (GCP), relevant regulations and standard operating procedures. The CI and PI, with the support of the Project Management Group (PMG) will be responsible for the day-to-day smooth running of the trial at the site. They will encourage recruitment, provide staff education and training, and monitor data completeness and quality. NPEU CTU will perform a trial risk assessment prior to commencement that will be reviewed at regular intervals during the course of the trial. The degree of central and site monitoring will be outlined in a separate Monitoring Plan, developed in light of any risks identified in the risk assessment. Site monitoring will be carried out by a suitably qualified person independent of the study team.

## Trial Governance

### Project Management Group (PMG)

The trial will be supervised on a day-to-day basis by the Chief Investigator and (CI) Principal Investigator (PI) with the support of the PMG. This group reports to the Trial Steering Committee (TSC) which is responsible to the trial Sponsor.

- The PMG will meet regularly (at least monthly).
- The core PMG will consist of the following:
- NPEU CTU Director
- NPEU CTU Senior Trials Manager
- NPEU CTU Trial Co-ordinator
- NPEU CTU Statistician
- NPEU CTU Programmer
- Principal Investigator
- Chief Investigator
- Principal Research Scientist
- Principal Clinical Researcher

### Trial Steering Committee (TSC)

The trial will be overseen by the TSC consisting of an independent chair and at least two other independent members.

### Data Monitoring Committee (DMC)

A DMC independent of the applicants and of the TSC will review the progress of the trial after every 25 patients have been randomised and provide advice on the conduct of the trial to the TSC and (via the TSC) to the PMG. The DMC will act according to its Charter, which will be agreed at its first meeting.

The aims of the DMC will include:

• To pick up any trends, such as increases in un/expected events, and take appropriate action
• To seek additional advice or information from investigators where required
• To evaluate the risk of the trial continuing and take appropriate action where necessary.

The DMC will monitor Safety outcomes very closely. Drug safety will be assessed by measuring the number of occurrences of apnoea that result in increase in respiratory support modality or require intervention via *NeoPuff* or ‘bag and mask’ or hypotension that require treatment with inotropes in the 24-hour period after drug administration. The DMC will review trial safety outcomes after every 25 patients have been randomised and safety data collected (i.e. n=25, 50, 75, 100 and 125). In addition, the Chief Investigator (or suitably trained delegate) will be notified of every such occurrence.

A formal sequential safety procedure will be applied and presented to the independent Data monitoring committee (DMC). We will employ a stopping boundary using group sequential methods with boundaries agreed by the DMC and specified in the DMC Charter. At each safety review performed by the DMC, a graph showing evidence of the relative safety of the treatments will be provided to act as a guide to the DMC members. If the pre-specified boundary is crossed, then the DMC should consider terminating the trial, taking into account other considerations (e.g. implications on future patients/clinical practice and follow-up, how convincing the evidence is, etc.).

An interim statistical analysis of the primary and secondary outcomes will be conducted once data has been collected from 50% of trial participants (78 infants). In the light of the interim data analysis, and other evidence from relevant studies (including updated overviews of relevant randomised controlled trials), the DMC will inform the TSC, if in their view there is proof beyond reasonable doubt that the data indicate that any part of the protocol under investigation is either clearly indicated or contra-indicated. Guidelines for early cessation will be agreed with the DMC and documented in the DMC Charter.

## Serious Breaches

The Medicines for Human Use (Clinical Trials) Regulations contain a requirement for the notification of "serious breaches" to the MHRA within 7 days of the Sponsor becoming aware of the breach.

A serious breach is defined as “A breach of Good Clinical Practice (GCP) or the trial protocol which is likely to affect to a significant degree –

(a) the safety or physical or mental integrity of the subjects of the trial; or
(b) the scientific value of the trial”.

Incidents and protocol deviations will be reported as soon as possible to the NPEU CTU and assessed by the PMG. In the event that a serious breach is suspected the Sponsor must be contacted within 1 working day. In collaboration with the CI, the serious breach will be reviewed by the Sponsor and, if appropriate, the Sponsor will report it to the REC committee, Regulatory authority and the NHS host organisation within seven calendar days.

## Ethical and Regulatory Considerations

### Declaration of Helsinki

The Chief Investigator will ensure that this study is conducted in accordance with the principles of the Declaration of Helsinki.

### Guidelines for Good Clinical Practice

The Chief Investigator will ensure that this study is conducted in full conformity with relevant regulations and with the Guidelines for Good Clinical Practice.

### Approvals

The trial will start after gaining approval from the MHRA and a Research Ethics Committee. Additionally, research governance approval of the host institutions will be sought.

The CI or their delegate will submit and, where necessary, obtain approval from the above parties for any substantial amendments to the original approved documents.

### Trial sponsor

The University of Oxford is the nominated sponsor for the trial.

## Finance and Insurance

### Funding

The Wellcome Trust and National Institute of Health Research, EME Programme is funding the trial.

### Insurance

The University has a specialist insurance policy in place which would operate in the event of any participant suffering harm as a result of their involvement in the research (Newline Underwriting Management Ltd, at Lloyd’s of London). NHS indemnity operates in respect of the clinical treatment which is provided.

## Publication Policy/Acknowledgement of Contribution

The Principal Investigator will co-ordinate dissemination of the results from this study. To safeguard the scientific integrity of the trial, data from this study will not be presented in public before the main results are published without the prior consent of the TSC.

The success of the trial depends on a large number of neonatal nurses, neonatologists, and parents. Credit for the study findings will be given to all who have collaborated and participated in the study.

All publications and presentations relating to the study will be authorised by the TSC. The first publication of the trial results will include as named authors, the Investigators, Statistician and Trial Co-ordinator. Members of the TSC and the DMC will be acknowledged and cited by name if published in a journal where this does not conflict with the journal’s policy. Authorship of secondary publications will be approved by the PI but will acknowledge the contribution of the TSC and the NPEU CTU.

Parents will be sent a summary of trial results; this will contain a reference to the full publication. A copy of the journal article will be available on request from NPEU CTU.
