# Supplementary material for: A blinded randomised placebo-controlled trial investigating the efficacy of morphine analgesia for procedural pain in infants: Trial protocol
Source: Wellcome Open Res. 2017 Jan 26;1:7. Originally published 2016 Nov 15. [Version 2] doi: 10.12688/wellcomeopenres.10005.2 (PMC5218543; doi:10.12688/wellcomeopenres.10005.2)
Supplement: Peer review history

# Peer review: Referee response for version 2

**DOI:** 10.21956/wellcomeopenres.11557.r19772

**Published:** 2017-01-31

**Authors:** Dick Tibboel

**Affiliations:** 1 Department of Pediatric Surgery / ICU, Erasmus MC-Sophia Children's Hospital, Rotterdam, 3015 CN, Netherlands

I understand the arguments by the authors. I feel that the approach using fentanyl is much more straight forward and directed to the solution of this problem. As a consequence I feel as the authors suggest themselves that a few sentences should be added to the manuscript regarding the effect and evaluation of short working opoids or opioid-like subtances.

 Overall I agree with the comments and with this additional information the manuscript can be accepted as such.

I have read this submission. I believe that I have an appropriate level of expertise to confirm that it is of an acceptable scientific standard.

# Peer review: Referee response for version 1

**DOI:** 10.21956/wellcomeopenres.10781.r19355

**Published:** 2017-01-16

**Authors:** Dick Tibboel

**Affiliations:** 1 Department of Pediatric Surgery / ICU, Erasmus MC-Sophia Children's Hospital, Rotterdam, 3015 CN, Netherlands

This protocol describes a blinded randomised placebo-controlled trial investigating the efficacy of morphine analgesia for procedural pain in infants.

 In the description of the protocol all the elements are appropriately addressed but the main underlying question is whether there is validity in taking such an approach.

 Literature data even today have shown that preterms in the ICU are submitted to on average 10 painful events without appropriate procedural analgesia.

 To this effect a number of procedures have been evaluated as recently by Carbajal *et al* using an enquiry in many neonatal intensive care units and the use of analgesic and sedative drugs as published in the Lancet 2015.

 There is a significant variability in the use of different analgesics and the optimal analgesic so far is not available for practice in daily NICU care.

 Apart from a variety of non-pharmacological approaches, including swaddling and kangarooing, pharmacological approaches can be divided in opioid and non-opioids while the latter can either be used as long or short working analgesic drugs.

 Morphine is a classic example of a drug that does not have an immediate effect and as such the trial design is appropriate as the drug is given about 1 hour before the procedure.

 This kind of approach has been used before for endotracheal intubation based on the concept that intubation is a painful event which is mainly derived on experiences and questionnaires from adults.

 Given the pharmacokinetic characteristics of morphine its mode of action and the slow time of onset it is very questionable whether this approach of evaluating oral morphine for procedural pain in infants is of value for our understanding and will change our attitude towards procedures like evaluation of retinopathy of prematurity (ROP screening and heel lancing).

 In fact a short working opioid related substance like fentanyl of one of the modern forms of fentanyl like- substances needs a higher urgency of evaluation than going back to a classic drug like morphine of which many clinicians will not be considering to give it 1 hour before a screening procedure.

 Moreover there is a lack of data on the oral consumption and absorption of morphine which can lead to a significant feeling of doubt of the relevance of this approach.

 Although I appreciate the detailed way of the protocol description in which all the elements are nicely documented and reported in a structured way the fundamental question whether this research is relevant for this vulnerable group of patients is arguable.

 My opinion is supported by 2 experts in evaluation of pain the newborn as well as a neonatologist – clinical pharmacologist who I consulted within this process of writing this comment.

I have read this submission. I believe that I have an appropriate level of expertise to confirm that it is of an acceptable scientific standard, however I have significant reservations, as outlined above.

# Response

**Published:** 2017-01-18

**Authors:** Rebeccah Slater

**Affiliations:** University of Oxford

We would like to thank Dr Tibboel for reading and reviewing our Trial Protocol. In his report Dr Tibboel suggests that although the study is well designed and described, he is not convinced that a study of this type is warranted to test the analgesic efficacy of morphine for procedural pain in prematurely born infants. We strongly disagree with this sentiment. If the results of this clinical trial were to show that the administration of a single oral bolus dose of morphine prior to retinopathy of prematurity screening (ROP) decreases noxious-evoked bran activity, reduces clinical pain scores, and prevents physiological instability that is reported to occur in the 24-hour period post ROP screening (characterised by an increase in the rates of apnoea, tachycardia, bradycardia and oxygen desaturation), there would be a strong rationale for its use in clinical practice. This is particularly important given that current treatment options for this procedure are not considered to provide adequate analgesia. Furthermore, if the administration of oral morphine prior to painful procedures is shown to be effective, it may also be useful for other painful procedures, and would increase the treatment options available to infants. The fact that it would need to be given an hour before a scheduled procedure should not negate its value. While morphine may be a ‘classic drug’ that is commonly administered to ventilated infants in intensive care, its primary use in this population is as a sedative and there is still controversy regarding its analgesic efficacy (as described in Dr Carbajal’s Referee Report). If the dose proposed here is effective this will also add considerably to the limited dosing data available for oral morphine. Furthermore, inclusion of our more recently developed measures of pain-related brain activity and the detailed physiological monitoring will add greatly to our mechanistic understanding of how pharmacological analgesics alter brain activity and physiological stability in prematurely-born infants. While we agree that it would also be valuable to use a similar approach to test the analgesic efficacy of fentanyl (which we are in the process of setting up), this does not negate the importance of testing the analgesic efficacy of morphine for procedural pain.

# Peer review: Referee response for version 1

**DOI:** 10.21956/wellcomeopenres.10781.r18752

**Published:** 2016-12-23

**Authors:** Ricardo Carbajal

**Affiliations:** 1 Center for Research on Epidemiology, Paris-Descartes University, Paris, France

This is the protocol of a blinded, placebo-controlled, parallel-group randomized, phase II, clinical trial that will be undertaken to determine whether morphine sulphate administered orally prior to clinically-required retinopathy of prematurity (ROP) screening and heel lancing provides effective analgesia. A total of 156 infants between 34 and 42 weeks’ corrected gestational age who require a clinical heel lance and ROP screening on the same test occasion will be included in the trial. Infants will be randomised to receive either a single dose of morphine sulphate (100 μg/kg) or placebo. Each infant will be monitored for 48 hours and safety data will be collected during the 24 hours following drug administration. The primary outcome will be the Premature Infant Pain Profile–revised (PIPP-R) score during the 30-seconds period after ROP screening. The co-primary outcome will be the magnitude of nociceptive-specific brain activity evoked by a clinically-required heel lance.

 This is a very relevant study because it aims at finding an effective analgesic strategy to reduce pain during ROP screening since no analgesic approach so far published seems adequate. Furthermore, it will contribute to obtain evidence of the analgesic efficacy of morphine in the neonate for procedural pain. Conflicting evidence exists about the efficacy of continuous morphine to relieve pain from routine procedures in ventilated preterm neonates. Two studies investigated their responses to tracheal suctioning ^1,2^; in one of them, Anand *et al *found a reduction in pain scores ^2^, whereas Simons *et al*, judging from pain scores obtained with three validated scales, found no difference between morphine and placebo groups ^1^. Pain measured by facial expression was diminished by morphine infusions during heel stick in preterm and term neonates, but pain measures did not correlate with plasma morphine concentrations ^3^. In another study, morphine given as a loading dose followed by continuous intravenous infusions to less than 48-hours old ventilated neonates under 33 weeks gestation did not appear to provide adequate analgesia for the acute pain caused by a heel stick ^4^. Thus, the use of two different procedures as well as the use of two different ways of assessing pain in the current study will contribute to a better understanding of the analgesic efficacy of morphine in neonates. Of note, at the time of the study intervention, neonates will be at least two weeks old and between 34 and 42 corrected gestational age. The close follow-up during 48 hours after the painful procedure is a major asset.

 The protocol is very well written and interventions are very clearly and repeatedly explained. The background section provides enough information to understand the importance of this study and the rationale of using these two procedures. The trial design section shows in the text and in the corresponding figure a clear outline of planned interventions. The safety monitoring section is complete and gives clear indications of the assessments and reporting of adverse effects. The statistical analyses are well presented and data management procedures seem very relevant.

**Specific comments on sections**

*Background and rationale*

 Page 3. The explanation of the distress caused by ROP needs, in my opinion, to be clarified. When citing Mitchell *et al *(Ref 14), it is said that the level of clinical distress caused by this procedure is exemplified in a recent study, which reports that in the 24-hour period following ROP screening 41% of infants have apneic episodes compared to only 19% after the examination. In fact, this information does not seem to correspond to what is mentioned in the article which indicates an apnea frequency of 12.8% before eye examination, 23.07% within the first 24 hours after eye examination (p=0.13) and 30.7% from 25-48 hours after eye examination (p=0.04).

 Page 3. If the described plausibility that the administration of morphine prior to ROP screening may provide effective pain relief and consequently reduce the resultant physiological instability caused by the procedure is true, one interpretation would be that this procedure elicits a long lasting pain because of the tissue damage or inflammation that may be created by the long lasting insertion of a speculum that pulls apart the eyelids.

 Page 4. While the use of nociceptive-specific brain activity may allow identifying patterns of nociceptive brain activity, it should be kept in mind during the discussion and interpretation of results that pain is a very complex phenomenon that could not be reduced to a very short lasting electrical activity. In a previous paper of the authors, this brain activity that lasted less than 100 milliseconds was used as a primary outcome to assess the analgesic efficacy of an oral sweet solution. In the current study, the authors will use again an immediate nociceptive electrical signal during a heel lance but also measure pain with a pain scale after the end of ROP screening. These two types of assessments seem complementary.

 Page 4. In the justification of the trial, the statement on the use of a dose of 200 µg/kg of oral morphine does not correspond to the cited reference (Ref 37).

*Trial design*

 Page 5. In box 1 and throughout the text, the description of the moment of the pain assessment for ROP screening is a bit confusing. It is said that pain will be assessed with the PIPP-R score 30 seconds after ROP screening. It would be more appropriate to say that pain will be assessed during the 30-seconds period after ROP screening.

 Page 5. Box 1, secondary outcome measures. The measurement of the incidence of apnea is only relevant for non-intubated infants. This should be clarified because nowhere in the protocol it is indicated that intubated infants will not be included. The use of an inotrope as a marker of hypotension requiring intervention is in my opinion insufficient because in many occasions the first intervention for morphine-induced hypotension will be volume replacement. Thus, this volume replacement should also be monitored and reported.

 Page 6. Inclusion-exclusion criteria. It should be clarified that intubated and non-intubated infants will be included.

 Page 7. Figure 2. In the exclusion criteria box, correct “…born to mothers who regularly used opiates…”

 Page 11. Reflex withdrawal. Reference 38 does not correspond to this statement; it should be reference numbered 37.

 Page 11. Safety of administration of oral morphine. As indicated above the incidence of apnea is only relevant to non-intubated infants.

 Page 12. Concomitant medications. The assertion “At present infants do not receive any pain medication prior to heel lancing so it is acceptable that the control group receive a placebo (inactive solution) with no medicinal effects.” Is not true everywhere. Indeed, the opposite is becoming true. Guidelines for managing painful procedures in different countries indicate the use of sucrose for heel lance. For instance a joint position statement from the Canadian Paediatric Society and the American Academy of Pediatrics recommend that a combination of oral sucrose/glucose and other nonpharmacological pain reduction methods (nonnutritive sucking, kangaroo care, facilitated tuck, swaddling and developmental care) should be used for minor, routine procedures ^5^; this recommendation was ratified in 2016 ^6^. In the UK, The Royal College of Anaesthetists recommends, in its Core standards for pain management services in the UK, the use of sucrose for painful procedures in infants.

 In conclusion, this is a very interesting and relevant protocol that addresses an important issue in neonatal pain management. It should contribute to the improvement of analgesic practices in this vulnerable population.

**References**

  

1.  Simons, S.H., et al., *Routine morphine infusion in preterm newborns who received ventilatory support: a randomized controlled trial.* Jama, 2003. **290**(18): p. 2419-27.
2. Anand, K.J., et al., *Analgesia and sedation in preterm neonates who require ventilatory support: results from the NOPAIN trial. Neonatal Outcome and Prolonged Analgesia in Neonates.* Arch Pediatr Adolesc Med, 1999. **153**(4): p. 331-8.
3. Scott, C.S., et al., *Morphine pharmacokinetics and pain assessment in premature newborns.* J Pediatr, 1999. **135**(4): p. 423-9.
4. Carbajal, R., et al., *Morphine does not provide adequate analgesia for acute procedural pain among preterm neonates.* Pediatrics, 2005. **115**(6): p. 1494-500.
5. Batton, D.G., K.J. Barrington, and C. Wallman, *Prevention and management of pain in the neonate: an update.* Pediatrics, 2006. **118**(5): p. 2231-41.
6. American Academy of Pediatrics, *Prevention and Management of Procedural Pain in the Neonate: An Update.* Pediatrics, 2016. **137**(2): p. 1-13.

I have read this submission. I believe that I have an appropriate level of expertise to confirm that it is of an acceptable scientific standard.

# Peer review: Referee response for version 1

**DOI:** 10.21956/wellcomeopenres.10781.r18679

**Published:** 2016-12-21

**Authors:** Karel Allegaert

**Affiliations:** 1 Department of Development and Regeneration, KU Leuven, Leuven, Belgium

This is a very well described study design to evaluate the impact of morphine administration on the pain expression during both ROP screening and heel lancing. Also the additional procedures (how the screening and lancing will be performed, additional interventions like swaddling, dilatation eye drops and local anaesthetics) are well described. This study design has the potential to provide an answer on the potential relevance of morphine for both procedural interventions after these ‘conventional interventions’ but alternative approaches (eg. venepuncture instead of heel lancing, or using less painful procedures of ROP screening or not considered).

I have read this submission. I believe that I have an appropriate level of expertise to confirm that it is of an acceptable scientific standard.
